# Degradation characteristics of 3D printed continuous fibre-reinforced PA6/chopped fibre composites in simulated saltwater

**DOI:** 10.1007/s40964-024-00654-5

**Published:** 2024-05-16

**Authors:** Pouyan Ghabezi, Tomas Flanagan, Michael Walls, Noel M. Harrison

**Affiliations:** 1 College of Science and Engineering, University of Galway, Galway, Ireland; 2 I-Form, the SFI Research Centre for Advanced Manufacturing, Dublin, Ireland; 3 Ryan Institute for Environmental, Marine and Energy Research, University of Galway, Galway, Ireland; 4 Éire Composites Teo, Údarás Industrial Estate, An Choill Rua, Inverin, Co., Galway, Ireland; 5 CTL Tástáil Teo, Údarás Industrial Estate, An Choill Rua, Inverin, Co., Galway, Ireland

**Keywords:** Aging, 3D printing, Seawater, Onyx

## Abstract

This paper investigates the performance of continuous fibre-reinforced 3D printed components in salt water medium at room temperature. Markforged^®^ Mark Two 3D printer was employed to fabricate standard specimens made of Onyx and reinforced Onyx specimens with continuous carbon, high-strength high-temperature glass, and Kevlar fibres. Aging process was conducted to characterize the long-term effect of salt water on the mechanical behaviour of fibre-reinforced 3D printed samples. Several mechanical tests including tensile, 3-point bending test and indentation testing have been carried out on the dry and aged standard samples to evaluate tensile strength, flexural strength, micro-hardness, and modulus of elasticity in micro-scale. The mechanical tests revealed the degradation and loss in mechanical properties of the printed samples after aging in salt water. The data highlighted that Onyx samples without continuous fibres experienced the most significant reduction in both tensile (33.54%) and flexural (63.47%) strengths after 1 year, while continuous carbon fibre-reinforced Onyx samples showed comparatively lower strength reductions (28.46% in tensile strength and 18.73% in flexural strength). Optical and scanning electron microscopy were performed to investigate the fracture behaviour of the tested specimens. In addition, the DSC assessment showed a slight change in the thermal properties of aged specimens.

## Introduction

The aging process in salt water can have significant implications for the performance and durability of 3D printed components. When exposed to a saline environment, the salt water can permeate the structure of the printed parts, leading to various forms of degradation. One of the primary concerns is plasticization, where the water molecules infiltrate the polymer matrix, causing it to soften and lose its mechanical strength [1, 2]. This plasticization effect can result in dimensional changes, warping, and reduced load-bearing capacity of the components. Additionally, the presence of salt ions can induce chemical reactions, such as hydrolysis, that further degrade the material’s properties over time [3]. This combination of physical and chemical degradation mechanisms can compromise the structural integrity and functional performance of 3D printed components, making it crucial to carefully consider the materials and post-processing techniques to mitigate the effects of aging in salt water [4].

The additive manufacturing techniques have revolutionized polymers and composites production compared to the traditional methods like hand lay-up, VARTM, injection moulding, extrusion, and compression moulding historically dominated large-scale fabrication [5–7]. One of the widely used additive manufacturing techniques is Material extrusion (ME), often known as fused deposition modelling (FDM), because of its simplicity and advances in feedstock and printers in spite of their inherent drawbacks because of layer by layer nature [8]. An effective way to enhance the mechanical properties of ME printed thermoplastic in industry is adding fibres to the filaments. Effect of water uptake on the mechanical and physicochemical characterization properties of composite materials has been investigated by some researcher to predict their long-term life and analyses hydrothermal behaviour [9, 10]. Two different technologies have been introduced by Markforged for composite 3D printing; (1) 3D printing of micro-chopped carbon fibre reinforced nylon matrix filament (with Onyx commercial name) and (2) 3D printing of Onyx filaments reinforced with continuous fibres [11].

There are many ongoing efforts to overcome the technical and environmental challenges associated with the use of additive manufacturing in maritime applications. Recently, Oak Ridge National Laboratory collaborated with the U.S. Navy’s Disruptive Technology Lab to support their latest endeavour in 3D printing. Together, they developed a submarine hull using carbon fibre reinforced composites [12]. Al Seer Marine [13] utilized a custom Flexbot machine equipped with two robots on linear tracks (offering a build volume of 4 × 36 m) to produce a 3D printed water taxi, setting the Guinness World Records™ title for the largest 3D printed boat. Tanaruz [14] based in the Netherlands manufactures 3D printed boats made of reclaimed polypropylene (PP) with 30% glass fibre.

3D printing in the maritime industry extends beyond the production of end parts. The versatility of this technology makes it ideal for creating moulds and tooling. By leveraging 3D printing for mould and tool fabrication, shipbuilders can achieve faster prototyping, reduced lead times, and enhanced customization capabilities. Additionally, the ability to iterate designs rapidly allows for greater innovation and efficiency in the manufacturing process [15].

He et al. [16] quantified the negative impact of voids on 3D printed continuous fibre-reinforced polymer composites. The void content in Carbon Fibre/Polyamide 6 (CF/PA6) composites was measured using optical microscopy and microcomputed tomography. Compression molding was used as a benchmark to achieve minimum void content. The mechanical properties and interlaminar fracture toughness of the composites were evaluated. The study highlighted the importance of developing in-process techniques during 3D printing to reduce void content in continuous fibre composites, thereby expanding their practical applications. Hadi et al. [17] examined the impact of key process parameters, moisture content, and heat treatment profiles on the temperature development and mechanical properties of 3D printed PA6 reinforced with chopped glass fibres. They determined that elevating the printing temperature enhances tensile strength, whereas minimizing moisture content reduces voids and improves tensile properties of 3D printed neat PA6 filament. Additionally, incorporating glass fibre into PA6 enhances tensile strength, exhibiting a maximum 45% increase when moisture is minimized and printing temperature is set to 270 °C. Machar et al. [18] employed accelerated seawater aging to evaluate acrylic/glass fibre and modified acrylic/glass fibre composites alongside a conventional epoxy/glass fibre baseline. Mechanical properties (including tensile, flexural, and short beam) were evaluated both before and after aging to compare their performance. Fracture surfaces are analysed using electron microscopy to investigate the effects of water ingress on fracture propagation. Additionally, diffusion coefficients of the composites in seawater were contrasted, and shifts in glass transition temperatures were used to gauge plasticization effects. Cetin and Fossi [19] addressed the limited exploration of hygrothermal effects on adhesively bonded 3D printed components, despite prior research on metal and composite adherents. They experimentally assessed the mechanical strength of such joints under hygrothermal conditions, using various surfaces and adhesive types over different durations. Various composites with different stacking sequences of continuous glass fibre layers were created and exposed to a hygrothermal environment for up to 30 days [20]. Multi-scale morphological analysis was used to understand deformation and failure mechanisms. The results indicated that hygrothermal aging affected mechanical properties through a combination of mechanisms. Polyamide matrix crystallization and stress relief enhanced strength, while reduced interlayer bonding and damage to glass fibres and the matrix weakened the composites. Composites with separate continuous glass fibre layer distribution exhibited greater initial hygrothermal stability, which decreased over time due to gradual delamination between layers [20].

The degradation of 3D printed components in a salt water environment is a complex process influenced by several factors, including the material composition, printing parameters, and exposure duration [4]. The presence of voids and interfacial gaps in the printed structure can act as preferential sites for water absorption, accelerating the degradation process. The water molecules can break down the polymer chains, leading to a decrease in mechanical properties, such as tensile strength, flexural strength, and modulus of elasticity [9]. Additionally, the continuous exposure to salt water can promote the growth of microorganisms, causing biofouling and further deteriorating the material. To mitigate degradation, strategies such as post-printing treatments, surface coatings, or the use of more resistant materials can be employed [21]. Understanding the specific degradation mechanisms and developing appropriate mitigation strategies is crucial for ensuring the long-term performance and reliability of 3D printed components in salt water environments. Numerous studies have explored the degradation and water aging of composite materials, especially thermoset polymers, across diverse manufacturing methods and applications [1, 22]. The existing literature lacks comprehensive research on the long-term degradation of 3D printed specimens. This study addresses this gap by investigating the degradation of four different groups of 3D printed samples, pure Onyx (PA6 + chopped carbon fibre) and three Onyx composite reinforced with continuous glass fibre, Kevlar fibre, and Carbon fibre, when exposed to saltwater at room temperature. This is a complex task due to the inherent voids and infilled regions in 3D printing. A comprehensive assessment involving mechanical tests (tensile and 3-point bending), indentation testing to assess the modulus of elasticity and hardness in micro level, optical microscopy, and differential scanning calorimetry was conducted on dry and aged specimens printed by MarkForged Mark 2, a commercial composite 3D printer.

## Materials and methods

In this study, four types of 3D printed samples were fabricated using Onyx (nylon and micro-chopped carbon fibre—CF/PA6) as the matrix material, and reinforced with continuous carbon fibre, Kevlar fibre, and high-strength high-temperature (HSHT) glass fibre. The CF/PA6 (Onyx) composites were prepared with a typical fibre volume fraction of 10%, ensuring a uniform distribution of the fibres throughout the matrix. The Onyx filament itself exhibited a modulus of elasticity of 2.4 GPa and a tensile stress at yield of 40 MPa, as specified in the manufacturer’s datasheet [23]. Carbon fibre has a tensile strength of 800 MPa (with a flexural strength of 540 MPa, according to ASTM D7901). Kevlar fibre, on the other hand, has a tensile strength of 610 MPa (with a flexural strength of 240 MPa). Lastly, the HSHT glass fibre exhibits a tensile strength of 600 MPa (with a flexural strength of 420 MPa), as determined by ASTM D3039 [23]. By combining the strength and stiffness properties of these reinforcing fibres with the Onyx matrix material, it is expected that the resulting 3D printed composites will possess enhanced mechanical performance compared to the pure Onyx samples. The use of continuous fibres, along with the appropriate fibre–matrix interface, can effectively improve the tensile and flexural properties of the printed parts, making them suitable for a wide range of applications that require high strength and stiffness.

**Fig. 1:**
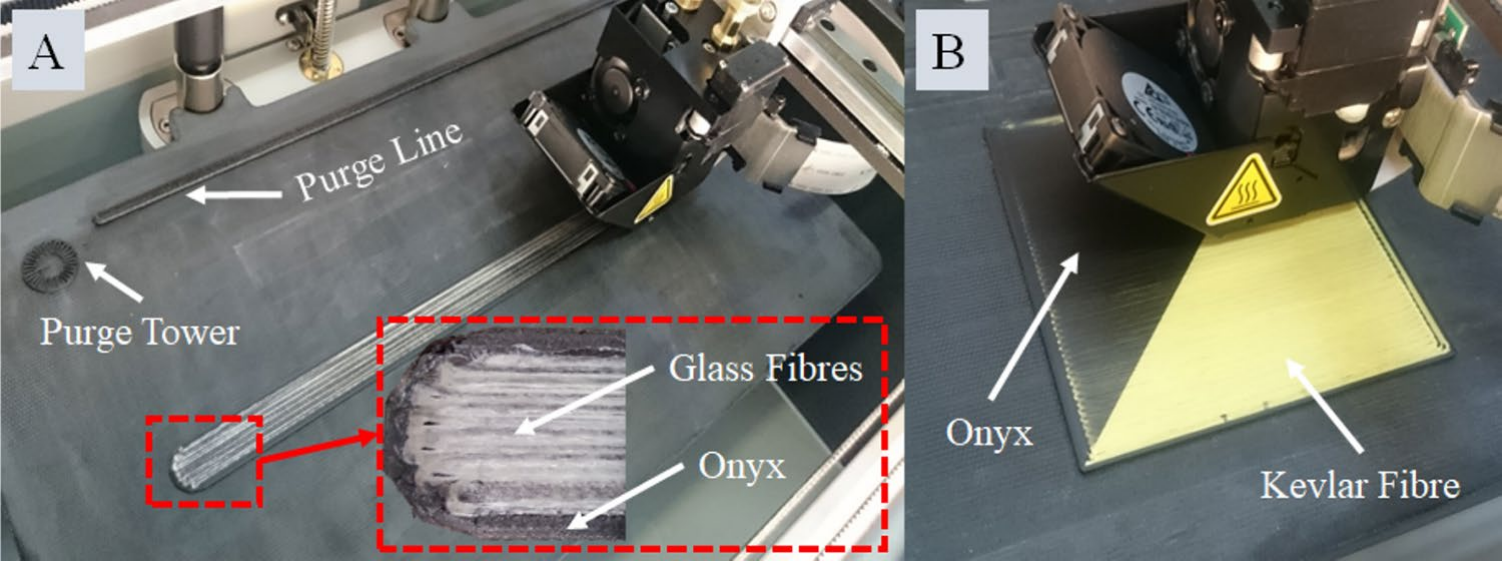
3D printed test specimens, **A** Onyx + Glass tensile test sample, **B** Onyx + Kevlar water uptake sample

All the specimens in this study were fabricated using a desktop 3D printer, specifically the Markforged^®^ Mark Two printer from the United States (Fig. 1). The printer’s capabilities allowed for precise and controlled printing of the composite materials. For the onyx samples and those reinforced with continuous carbon fibre, a layer height of 0.125 mm was chosen. This layer height ensured a good balance between printing speed and resolution, resulting in reliable and accurate printed parts. The reinforced samples with continuous Kevlar and HSHT glass fibres had a slightly lower layer height of 0.1 mm, providing even finer details and improved surface finish. The printing process involved controlling the temperatures of the matrix and fibre nozzles. The matrix nozzle temperature was set to 275 °C to achieve the optimal melting and flow properties of the onyx material. The fibre nozzle temperature, on the other hand, was automatically set to 250 °C for all the printed samples [24]. This temperature was carefully chosen to ensure proper bonding and integration between the matrix material and the continuous reinforcing fibres. It is worth mentioning that the raw materials used for printing, including the onyx material and the continuous carbon, Kevlar, and HSHT glass fibres, were supplied by the same company, Markforged^®^.

**Fig. 2:**
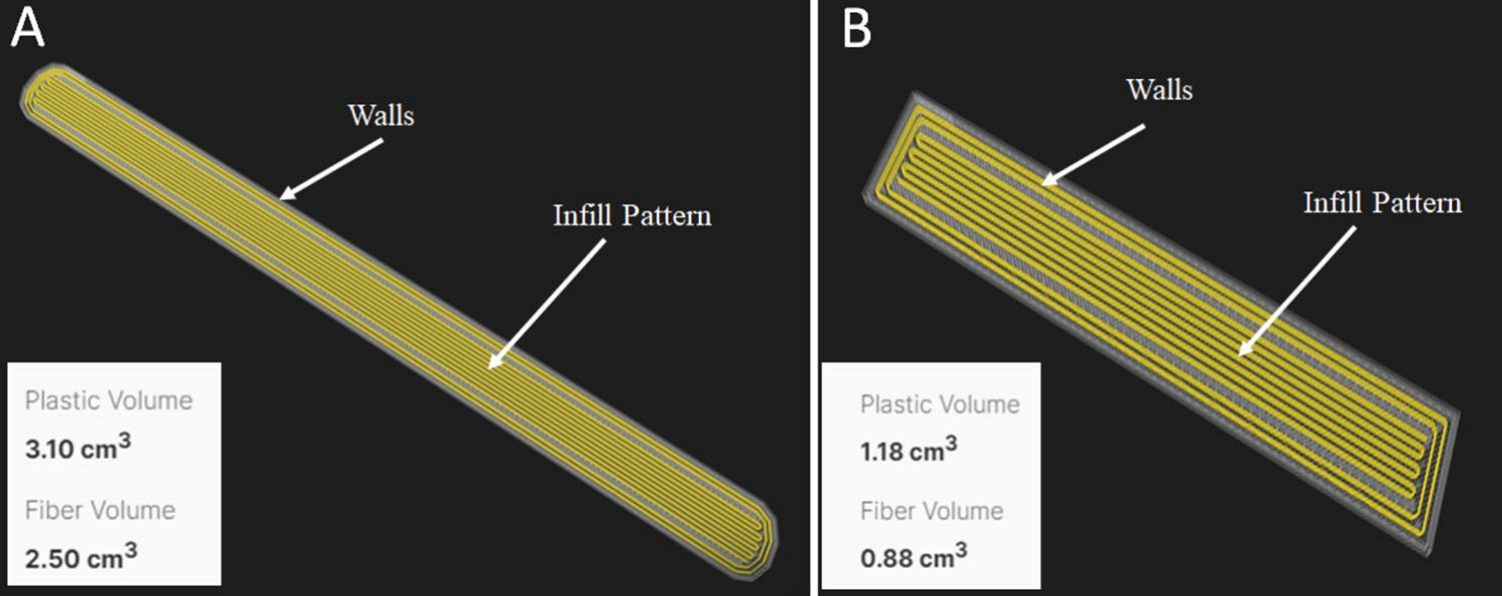
Fibre orientation and plastic and fibre volume for **A** Onyx/Kevlar tensile specimens, and **B** Onyx/Kevlar flexural specimens

For the tensile testing and three-point bending samples in this study, specific 3D printing configurations were chosen to ensure compliance with the relevant ASTM standards. Tensile testing was performed according to ASTM D3039 [25] for the reinforced samples and ASTM D638 [26] for the Onyx samples. Three-point bending tests followed the guidelines outlined in ASTM D7264 [27]. For each test result, five samples were tested and the average value is reported. To achieve the desired sample properties, the 3D printing process involved using a solid fill pattern with two wall layers. Additionally, the reinforced samples had an isotropic fibre fill type, with a fibre angle of 0°. It should be noted that while efforts were made to match the fibre volume fractions between the tensile and bending samples, exact replication was challenging due to the proprietary nature of the Markforged 3D printers used in this study. The continuous fibre volume fraction of the tensile Onyx-Carbon specimens was determined (based on the printer output) to be approximately 36.47% (35.15% in the bending sample). In the same way, it was calculated as 27.62% for the tensile and bending Onyx-HSHT Glass specimens. The continuous fibre volume fraction of the tensile Onyx-Kevlar was approximated at 44.64% [42.71% in the bending sample (Fig. 2)]. These volume fractions indicate the proportion of continuous reinforcing fibres present in the composite samples, contributing to their enhanced mechanical properties. Although achieving the exact same fibre volume fraction in both the tensile and bending samples was not possible due to the limitations of the Markforged 3D printers, efforts were made to maintain similar volume fractions. This allowed for a meaningful comparison of the mechanical performance between the different sample types and reinforced materials. By carefully selecting the appropriate 3D printing configurations and considering the fibre volume fractions, the fabricated samples were tailored to meet the requirements of the subsequent mechanical testing methods, providing valuable insights into the performance of the Onyx and continuous fibre-reinforced composites.

**Fig. 3:**
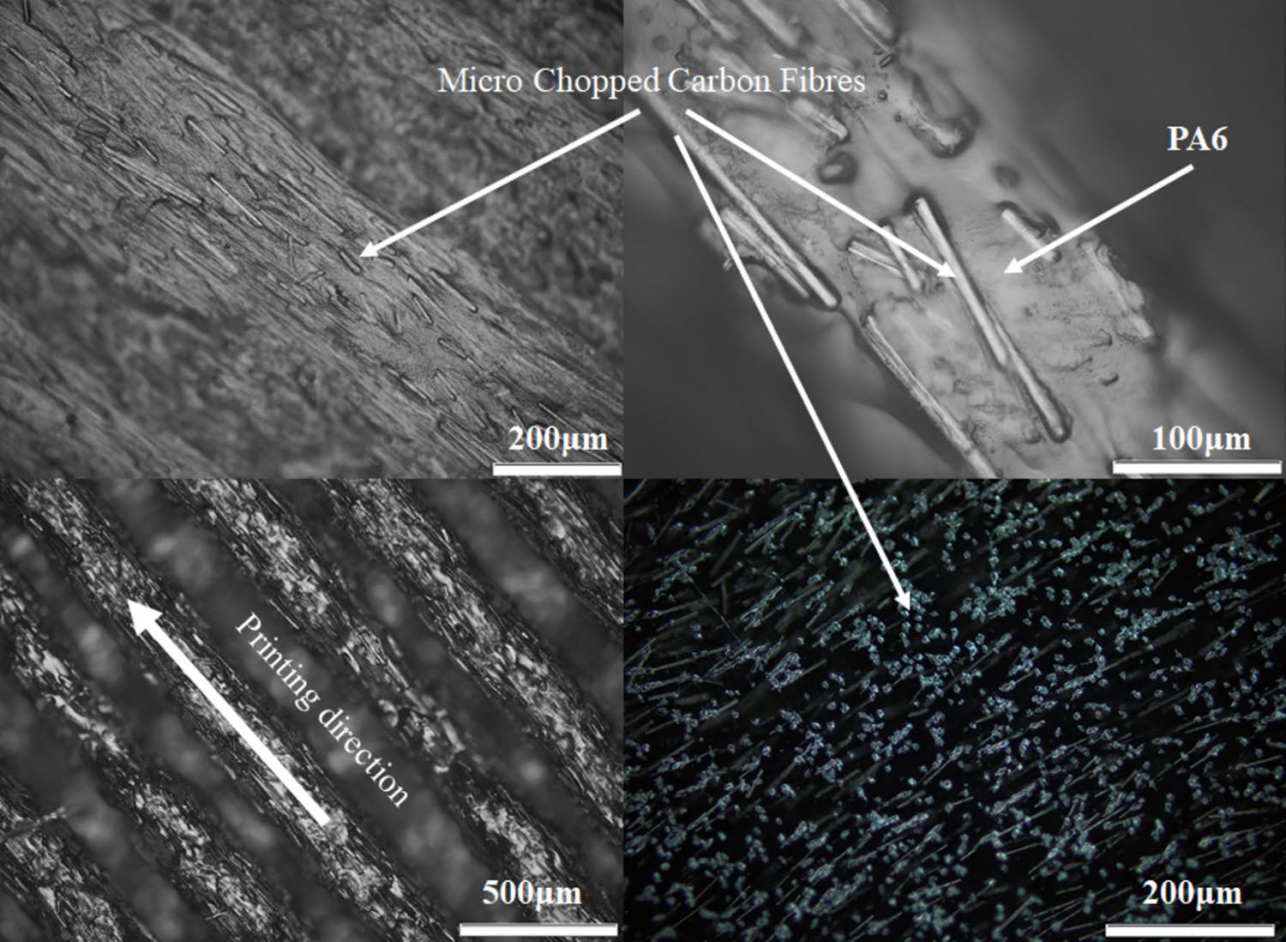
Microscopy images of printed samples

In order to evaluate the distribution and alignment of micro chopped carbon fibres in the printed parts, microscopy images were acquired using an Olympus BX51M light microscopy instrument equipped with a UC30 camera. These images provided valuable insights into the internal structure of the samples and allowed for a detailed assessment (Fig. 3). The microscopy analysis involved examining the cross-sectional views of the printed samples. By carefully studying these images, it was possible to confirm that the fibre volume fraction in the Onyx samples was approximately 10% as mentioned earlier. This volume fraction value indicates the proportion of fibres present in the Onyx material, providing a measure of the micro-chopped reinforcement content. The microscopy images not only validated the reported fibre volume fraction but also allowed for the visualization of the fibre distribution within the Onyx matrix. The micrographs revealed the presence of both micro chopped carbon fibres and continuous fibres, providing a clear understanding of their arrangement and orientation within the printed parts. This information is crucial for assessing the mechanical properties and performance of the composite materials. The utilization of light microscopy, coupled with the high-resolution capabilities of the UC30 camera, ensured that the acquired images captured the intricate details of the fibre distribution. These observations contribute to the overall understanding of the printed samples’ internal structure, enabling further analysis and interpretation of their mechanical behaviour.

The aging process of the printed samples was conducted under ambient conditions at room temperature (22 °C) in salt water with a salinity of 3.5%. In this study, elevated temperatures were not employed due to the relatively low glass transition temperature of Onyx materials, which is approximately 50 °C. According to ASTM D 5229/D, the aging temperature shall be at least 20 °C less than the glass transition temperature of the matrix [9]. To create the salt water solution, 35 g of table salt were added to a beaker, and tap water was then added until the total mass reached 1000 g. This solution was used for the aging process of the 3D printed specimens, which were immersed in the solution. For maintaining a continuous testing condition during the aging process, a Genlab Classic Unstirred Water Bath (Genlab Ltd, UK, Product code: EZ1) was utilized. This water bath provided a controlled environment for the specimens throughout the duration of the aging process.

The tensile strength analysis of 3D printed standard samples was carried out using an Instron 4467 machine, which was equipped with a 30kN load cell, in strict accordance with the ASTM D3039 guidelines. This meticulous testing process was designed to evaluate the mechanical properties of the samples under tensile loading conditions. The samples, each having a 2 mm thickness, were subjected to tensile testing both before and after they had undergone a saltwater aging process.

In addition to the tensile strength tests, the study also employed microscopy techniques to capture high-resolution images of the fracture surfaces of the tested specimens. This microscopy analysis allowed for an in-depth investigation of the fracture behaviour and morphology of the 3D printed materials after exposure to the saltwater aging process.

Flexural strength analysis was performed on both aged and dry printed samples according to ASTM D7254, employing a crosshead speed of 1 mm/min using an Instron 4467 machine.

This study aimed to investigate the microscale properties, specifically the modulus of elasticity and hardness, of dry and aged Onyx samples using the indentation technique. The experiments were conducted utilizing a Keysight Nanoindenter G200, adhering to ISO 14577 standards [28]. For the indentation experiments, a load control method with a standard load of 480 mN was employed. The hardness and modulus of elasticity were calculated at the maximum load of the indenter, with the average values obtained between the depths of 100 nm and 200 nm, respectively.

To investigate the thermal properties of Onyx 3D printed samples, a temperature scan was conducted using a DSC 214 Polyma system equipped with Proteus^®^ 7.0 software manufactured by NETZSCH. The samples were subjected to aging in salt water and then analysed from 40 to 250 °C at a heating rate of 20 °C/min. The airflow during the scan was maintained at 50 mL/min.

## Results and discussion

**Fig. 4:**
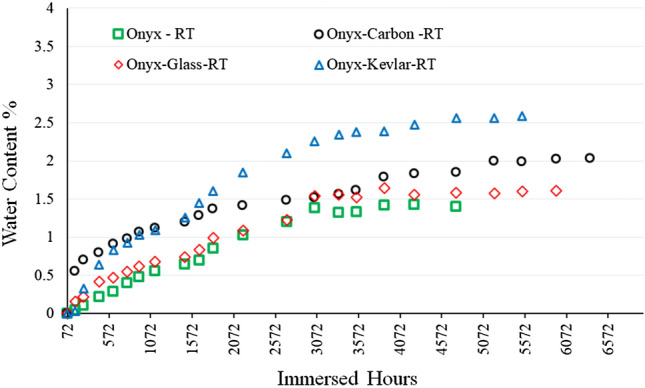
Water content absorbed in the printed specimens vs. immersion time

The study presented in Fig. 4 shows the water content in 100 mm × 100 mm samples made from Onyx, Onyx-Glass, Onyx-Carbon, and Onyx-Kevlar over time, following the ASTM D 5229/D 5229M standard. The results reveal the water absorption behaviour of these different materials and provide essential insights into their long-term durability and performance characteristics.

The water absorption curves were generated by monitoring the mass changes in all samples over time as they aged. The moisture content at each measurement point (*M*_t_) was determined using Eq. 1.

1 $$M_{{\text{t}}} \left( \% \right) = \left( {W_{{\text{t}}} - W_{0} } \right)/W_{0} \times 100,$$

where *W*_0_ and *W*_t_ are the initial weight and measured weight of the specimen, respectively.

It is evident that all the tested materials exhibit a gradual increase in water content as they are exposed to the ambient environment over time. This rise in water content is attributed to the materials’ ability to absorb moisture from the surrounding salt water, a phenomenon commonly referred to as hygroscopic behaviour and also the voids and unfilled areas occurred during printing. The Onyx-Kevlar and Onyx-Carbon samples reached their saturation points after a long duration, approximately 5204 h and 5952 h, and at a water content of 2.55% and 2.02%, respectively. In contrast, the Onyx and Onyx-Glass specimens reached their saturation points after approximately 3883 h (1.42% water content), and 4747 h (1.58% water content), respectively. This suggests that Kevlar and carbon reinforcements slow down the water absorption rate and increase the saturation point compared to the other materials in the study.

These results imply that the type and level of reinforcement have an impact on both the rate and extent of water absorption. It revealed that the fibre volume fraction and fibre type in the printed specimens effect on the water absorption behaviour of the studied samples (Table 1). The relationship between the continuous fibre volume fraction and the saturation point is straightforward. A higher continuous fibre volume fraction typically leads to a higher saturation point, indicating a greater ability to absorb moisture.

**Table 1:** Relation between the continuous fibre volume fraction and the saturation point in the 3D printed specimens

Materials	Continuous fibre volume fraction (%)	Saturation point (%)	Day to reach saturation point
Onyx	–	1.42	161
Onyx + Glass	27.62	1.58	197
Onyx + Carbon	36.47	2.02	248
Onyx + Kevlar	44.64	2.55	216

A coding system was incorporated to name the aged samples in this work (Material-aged temperature–time). Based on this system, Onyx + Kevlar-T22-180, means that the samples were made of Onyx reinforced with Kevlar aged in salt water at 22 °C for 180 days.

**Fig. 5:**
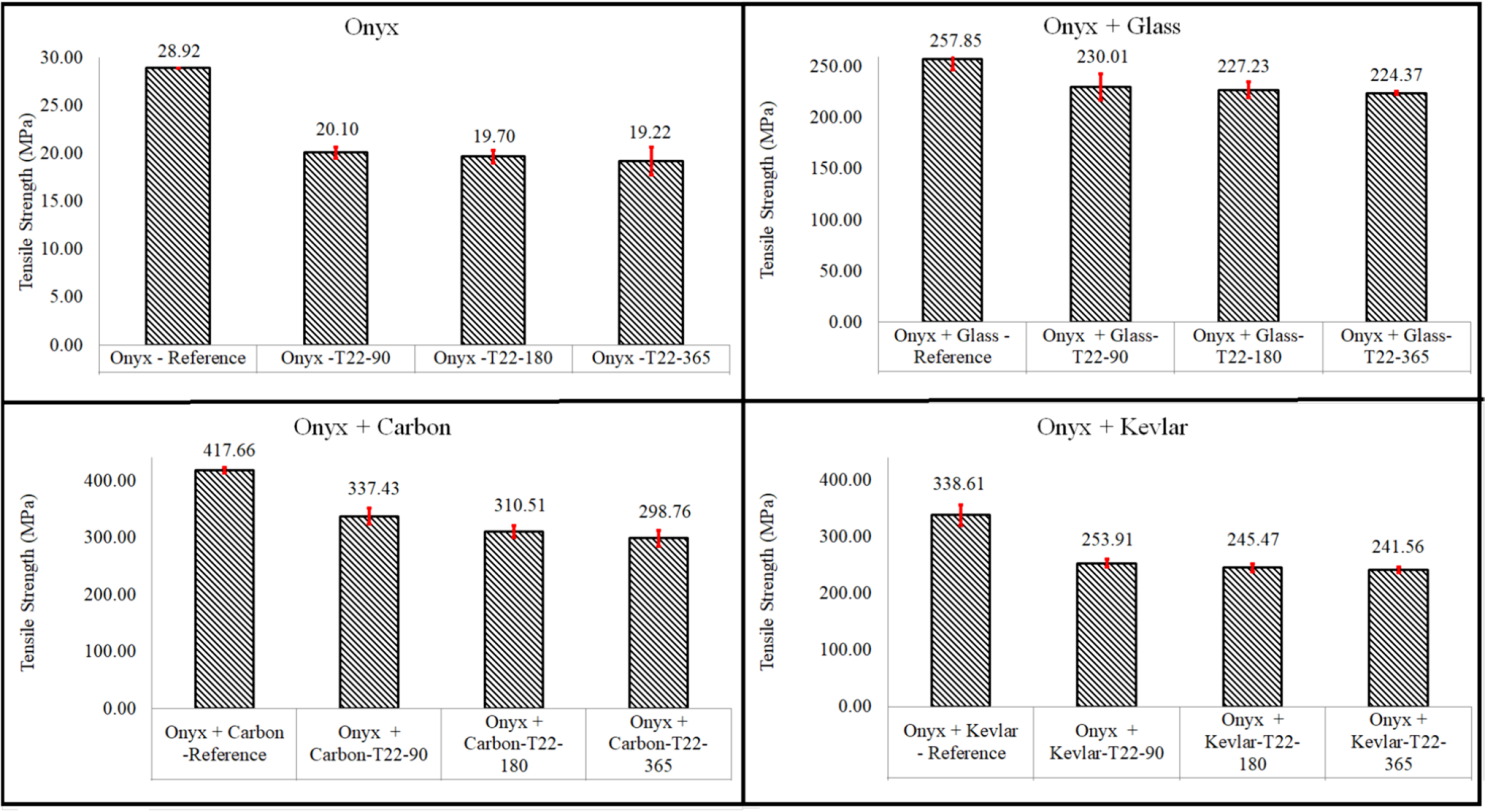
Tensile strength of un-aged/dry and aged samples

For the dry/Reference Onyx samples, the initial measured tensile strength was found to be 28.92 MPa. However, when subject to an aging process in saltwater at room temperature, the tensile strength of the Onyx samples exhibited a gradual and expected decline over time (Fig. 5). After a period of 90 days in the saltwater environment, the tensile strength value decreased to 20.1 MPa, indicating the material’s sensitivity to the prolonged exposure to moisture before reaching the saturation point (mostly mechanical degradation). This reduction in tensile strength over time suggests that the saltwater aging process has a significant impact on the mechanical properties of the material. Continuing the assessment, the tensile strength further decreased to 19.7 MPa after 180 days of immersion, and it decreased to 19.22 MPa after 1 year of aging (mechanical and chemical degradation).

The un-aged Onyx-Glass specimens exhibited an impressive tensile strength of 257.85 MPa, which gradually decreased to 230 MPa after 90 days of aging (before reaching the saturation point), further diminishing to 227.23 MPa after 180 days, and ultimately reaching 224.37 MPa after a full year of aging (after reaching the saturation point). Figure 5 visually demonstrates the profound influence of the aging process on the tensile strength of Onyx-Glass samples, emphasizing the material’s vulnerability to extended exposure to moisture. The dry Onyx-Carbon specimens displayed a tensile strength at 417.66 MPa. However, this strength was significantly reduced to 298.76 MPa after enduring 1 year of immersion in saltwater. The substantial decrease in tensile strength underlines the sensitivity of Onyx-Carbon composites to extended exposure to saline conditions. Similarly, the Onyx-Kevlar samples exhibited a decrease in tensile strength, transitioning from a robust 338.61 MPa in un-aged samples to 241.56 MPa in aged specimens after 365 days. This highlights the diminishing structural integrity of the material when subjected to prolonged exposure to saltwater.

The Onyx samples exhibited a significant 30% loss in their tensile strength after just 90 days of aging, while their tensile strength remained relatively stable even after a full year of saltwater exposure. On the other hand, the Onyx-Glass, Onyx-Carbon, and Onyx-Kevlar samples all experienced reductions in tensile strengths of 13%, 28.5%, and 28.7%, respectively, after 365 days of aging. It means that the degradation in mechanical properties continued even after reaching the saturation point.

These findings provide insights into the mechanical responses of additively manufactured composites under prolonged exposure to saltwater environments. Understanding the degradation trends in tensile strength is critical for assessing the long-term performance and durability of such materials in marine applications.

**Fig. 6:**
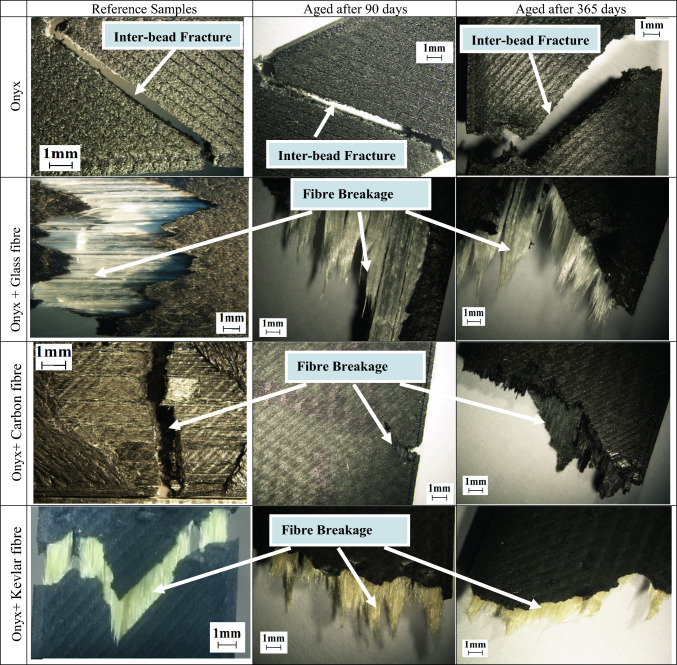
Microscopic images of fractured tensile samples of neat Onyx and composites

**Fig. 7:**
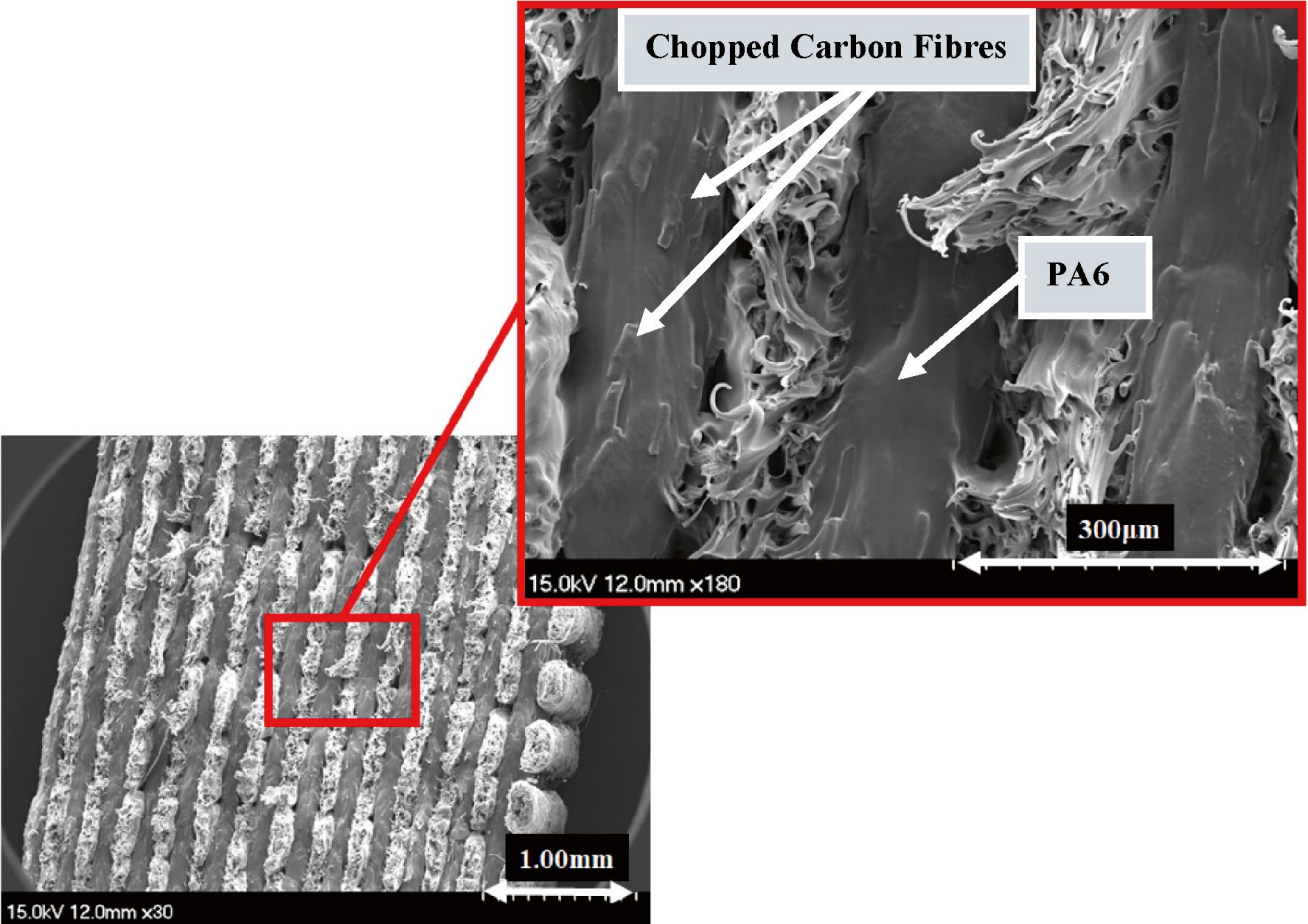
SEM micrographs for reference Onyx specimens

**Fig. 8:**
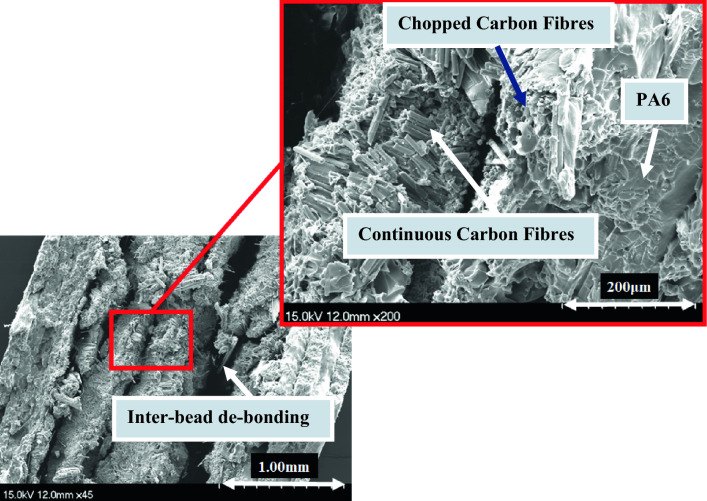
SEM micrographs for reference continuous fibre (carbon) reinforced Onyx specimens

**Fig. 9:**
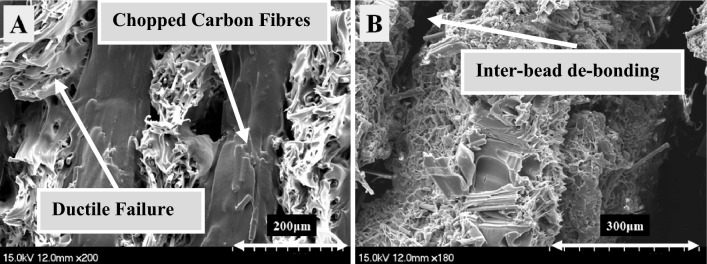
SEM micrographs for **A** Onyx specimens aged after 365 days, **B** continuous Carbon fibre reinforced Onyx specimens aged after 365 days

Figure 6 shows microscopic images of fractured tensile samples, Onyx, Onyx + Glass fibre, Onyx + Carbon fibre, and Onyx + Kevlar fibre composites. The figures clearly demonstrate the load-bearing capability of these materials. The enhancement in tensile strength and stiffness of the composites, as compared to neat Onyx, can be attributed to the presence of continuous fibres. Observing the images, it is evident that the failure mode in the Onyx samples remains unchanged throughout the aging process, occurring at the overlap of the printing beads. It is confirmed by the SEM images from the reference and aged Onyx specimens after 1 year (Fig. 7). However, in the samples reinforced with continuous Kevlar fibres and Glass fibres, the dominant failure modes involve fibre breakage and debonding between the fibres and the thermoplastic. In the images captured from samples aged over 90 days and 1 year, fibre breakage emerges as the primary failure mode. In the analysis of Figs. 8 and 9B, it becomes evident that the primary cause of complete fracture in Onyx + Carbon samples is the fibre breakage resulting from crack advancement.

This failure mechanism stands out as the most prominent factor contributing to the overall fracture process. SEM micrographs further illuminate the robust connection between the thermoplastic and the chopped carbon fibres, indicating a strong attachment that improves the fibber’s ability to resist micro-crack growth. In addition, the fracture resistant is evident through mechanisms such as continuous fibre breaking, where the fibre absorbs energy during the fracture process. The increase in tensile strength can be predominantly attributed to these effective crack resistance and energy absorption mechanisms.

The contrast in strength between Onyx + carbon composites and Onyx + Kevlar and Onyx + Glass composites can be attributed to the prevalence of fibre breakage as the primary failure mechanism in the former, as depicted in Figs. 8 and 9B. The firm attachment of the thermoplastic to carbon fibres enhances the composite’s ability to withstand crack propagation, resulting in a higher overall strength compared to Kevlar-reinforced composites. Moreover, Fig. 9A provides confirmation that the aging process, even after a year, does not significantly impact the ductile failure in the matrix of reference Onyx specimens (Fig. 7). The presence of continuous fibres in the 3D printed samples resulted in fibre breakage as the dominant failure pattern under tensile loading. The higher strength of the layers with continues fibres compared to those without fibres, considering the layer by layer nature of printed specimens, causes an inter-bead failure during loading as shown in Figs. 8 and 9. The inter-bead failure in these samples is different with the inter-bead failure observed in the non-continuous fibre reinforced samples (Fig. 6 for Onyx specimens). In the former, the failure happens between to bead beside each other from the same layer, while the inter-bead failure in the continuous fibre reinforced samples occurs between two layers (beads from up and down layers).

**Fig. 10:**
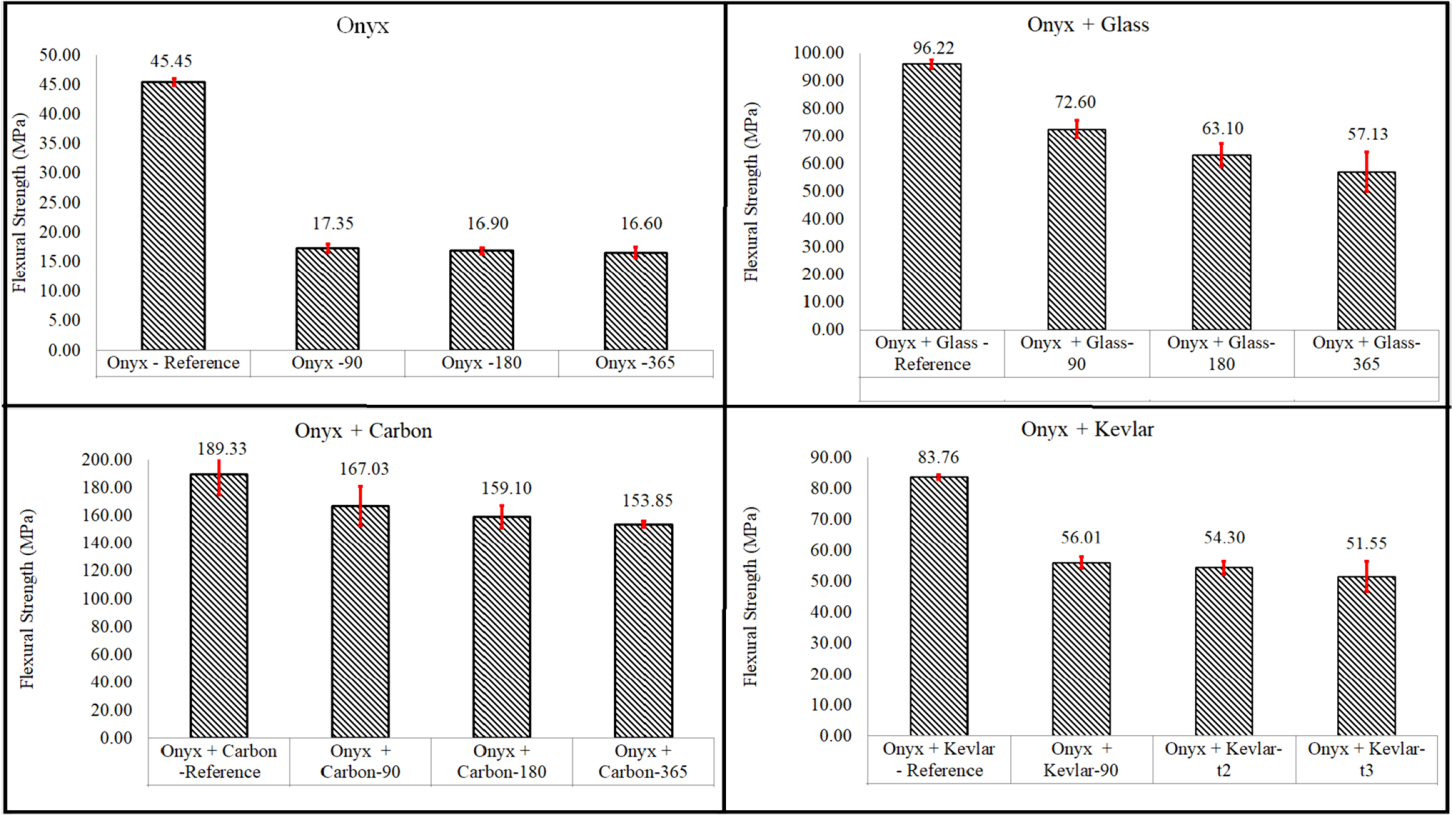
Flexural strength of 3D printed specimens

The results obtained from this investigation demonstrated distinct variations in flexural strength values for different durations of aging. The mean flexural strength values for dry Onyx samples, as well as those aged after 90, 180, and 365 days, were measured at 45.45 MPa, 17.35 MPa, 16.9 MPa, and 16.6 MPa, respectively (Fig. 10). When examining the flexural strength of Onyx-glass specimens, the experimental measurements showed mean values of 72.6 MPa, 63.1 MPa, and 57.13 MPa after 90, 180, and 365 days of aging, respectively, while the flexural strength of dry Onyx-glass samples was calculated to be 96.22 MPa. These results suggest a decline in flexural strength for the Onyx-glass specimens as a consequence of prolonged exposure to the aging environment. Furthermore, Fig. 10 depicts that the flexural strength of Onyx-carbon and Onyx-Kevlar printed specimens experienced reductions of approximately 18.73% and 38.45%, respectively, after 1 year of immersion in salt water when compared to their corresponding dry samples.

Significantly, Fig. 10 presents a significant decrease in the flexural strength of aged Onyx samples after 1 year, exhibiting a 63.5% drop compared to the dry Onyx sample. Similarly, Onyx-glass specimens experienced a considerable reduction of 40% in their flexural strength within the same time frame. The flexural strength losses for Onyx-carbon and Onyx-Kevlar specimens were recorded as 18.7% and 38.45%, respectively. The water uptake measurements (refer to Fig. 4) and the experimental mechanical strength results (shown in Figs. 5 and 10) have provided evidence of degradation in the printed samples, even after reaching the saturation point where water absorption ceased. These observations suggest that the chemical degradation of the Onyx matrix still occurs even when the maximum water content is reached, leading to a weakened interface between the continuous fibres and the Onyx material.

The investigation into the flexural strength behaviour of additively manufactured composites subjected to saltwater aging has revealed significant reductions in strength over time. The findings highlight the importance of considering the long-term durability and performance limitations of these composites in real-world environments. Future research efforts should focus on developing strategies to mitigate degradation and enhance the overall performance of additively manufactured composites in saltwater environments.

As the studied samples are continually exposed to a moist environment, the unavoidable water uptake sets off a chain of intricate processes that can profoundly affect the material’s properties. This includes plasticization, swelling, matrix hydrolysis, mass loss, chemical alterations, and the debonding of the fibre/matrix interface. All of these factors contribute to the gradual degradation of the mechanical properties of the materials.

The phenomenon of moisture absorption initiates an osmotic process within the composite materials. This osmotic process, in turn, leads to swelling, changes in interfacial adhesion between different layers, debonding of the fibre/matrix interface, and plasticization within the printed specimens [29]. These cumulative effects ultimately result in a significant reduction in the mechanical strength of the material.

Moisture diffusion within the printed samples plays a crucial role in these processes. It induces swelling, which, in turn, impacts the cohesive strength of the interface between different plies or layers within the composite. The weakening of this inter-ply interface significantly affects the overall strength of the material, leading to a decrease in its mechanical properties. The understanding of these complex interactions between moisture absorption, swelling, and interfacial adhesion is vital in evaluating the long-term performance and durability of materials, particularly in environments where exposure to moisture is a primary concern. This knowledge can inform strategies to mitigate the adverse effects of moisture and enhance the resilience of printed specimens in the face of challenging conditions.

The decline in mechanical strength during the aging process for both the printed Onyx and Onyx reinforced with continuous fibre samples is summarized in Table 2. According to the data in this table, the Onyx samples without continuous fibres exhibited the most significant reduction in both tensile strength (33.54% after 1 year) and flexural strength (63.47% after 1 year).

**Table 2:** Effect of aging time on the mechanical strength reduction of 3D printed specimens

	Samples	After 90 days	After 180 days	After 365 days
Tensile strength reduction %	Onyx	30.49	31.88	33.54
	Onyx + Glass	10.79	11.87	12.98
	Onyx + Carbon	19.2	25.65	28.46
	Onyx + Kevlar	25.01	27.5	28.66
Flexural strength reduction %	Onyx	61.81	62.81	63.47
	Onyx + Glass	24.55	34.42	40.62
	Onyx + Carbon	11.77	15.96	18.73
	Onyx + Kevlar	33.12	35.17	38.45

In contrast, the Onyx specimens reinforced with continuous fibres showed much lower strength reductions compared to the pure Onyx samples. Among the 3D printed samples reinforced with continuous fibres, the Onyx + Glass samples exhibited the least reduction in tensile strength (12.98%), while the Onyx + Carbon samples displayed the smallest reduction in flexural strength (18.73%). On the other hand, the highest reduction in tensile strength among the continuous fibre-reinforced Onyx samples was observed in Onyx + Kevlar and Onyx Carbon samples, both showing nearly identical reductions at approximately 28.5%. Meanwhile, Onyx + Glass samples exhibited the most substantial reduction in tensile strength (40.62%).

**Fig. 11:**
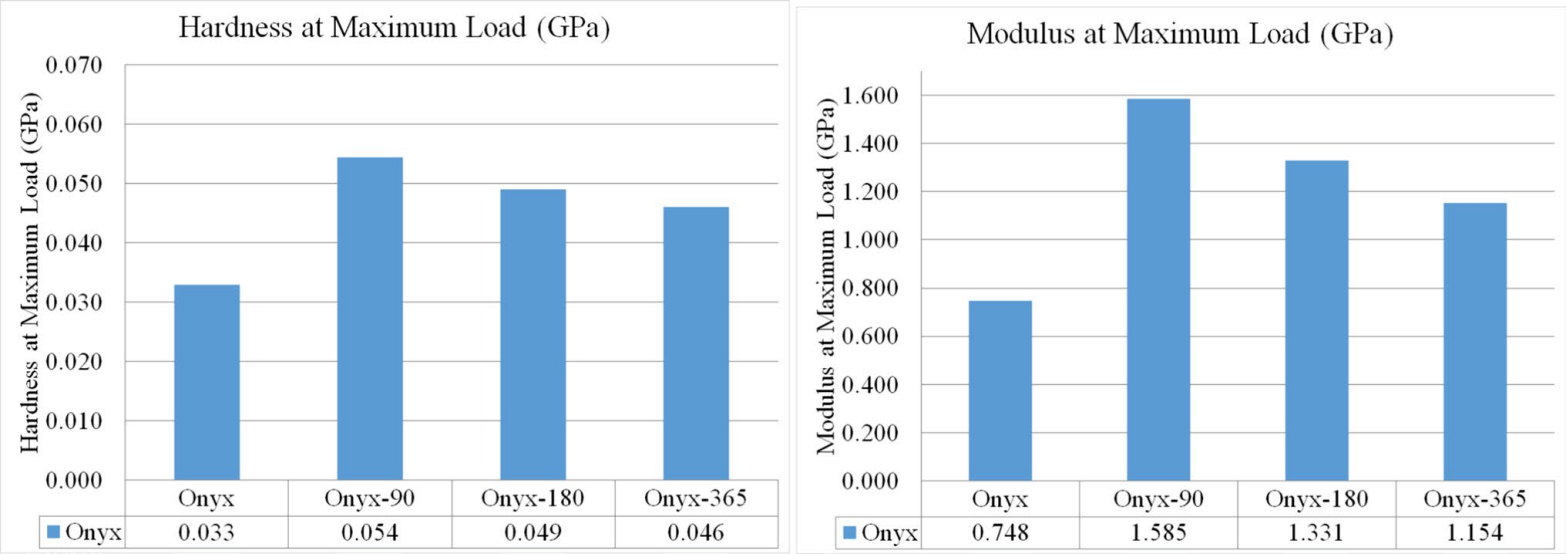
Hardness and Modulus of elasticity of the aged composites in micro level

The results obtained from the indentation testing provided valuable insights into the microscale properties of the investigated dry thermoplastic, Onyx (Fig. 11). The modulus of elasticity of the dry Onyx, determined using the standard indentation method, was measured as 0.748 GPa. Comparatively, the modulus of elasticity obtained from tensile testing was found to be 0.665 GPa, indicating a slight variation between the two testing methods. Furthermore, the effect of the aging process on the modulus of elasticity of the Onyx polymer was assessed. The modulus of elasticity at maximum load was measured as 1.585 GPa (0.535 GPa from tensile test), 1.331 GPa (0.466 GPa from tensile test), and 1.154 GPa (0.443 GPa from tensile test) after 90 days, 180 days, and 365 days of aging, respectively. These findings illustrate the impact of the aging process on the mechanical properties of Onyx (modulus of elasticity).

Interestingly, the experimental results reveal that the values for the elastic modulus at the microscale level were higher than those measured using the Instron machine at the macro-level. This discrepancy could be attributed to the inherent differences in the testing methodologies and the sensitivity of the indentation technique in capturing microscale variations in material properties. In addition to modulus of elasticity, the micro hardness at the maximum load was also determined through indentation testing. The dry Onyx exhibited a micro hardness of 0.033 GPa. However, after 90 days of aging, the hardness increased to 0.054 GPa. Similarly, the hardness values for Onyx aged after 180 days and 1 year in salt water were measured as 0.049 GPa and 0.46 GPa, respectively. These results highlight the effect of aging on the hardness properties of Onyx, indicating an increase in hardness over time. The increase in the hardness of the aged specimens indicate that the polymer became more brittle after reaction with salt water over the aging process. It is confirmed by the same results captured for the modulus of elasticity change over aging time (Fig. 11 Right).

Overall, the indentation technique provided valuable details for the microscale properties of Onyx, including modulus of elasticity and hardness. The results obtained through this method complement the findings from the macro-level tensile testing, further enhancing our understanding of the material’s mechanical behaviour and its response to the aging process.

**Fig. 12:**
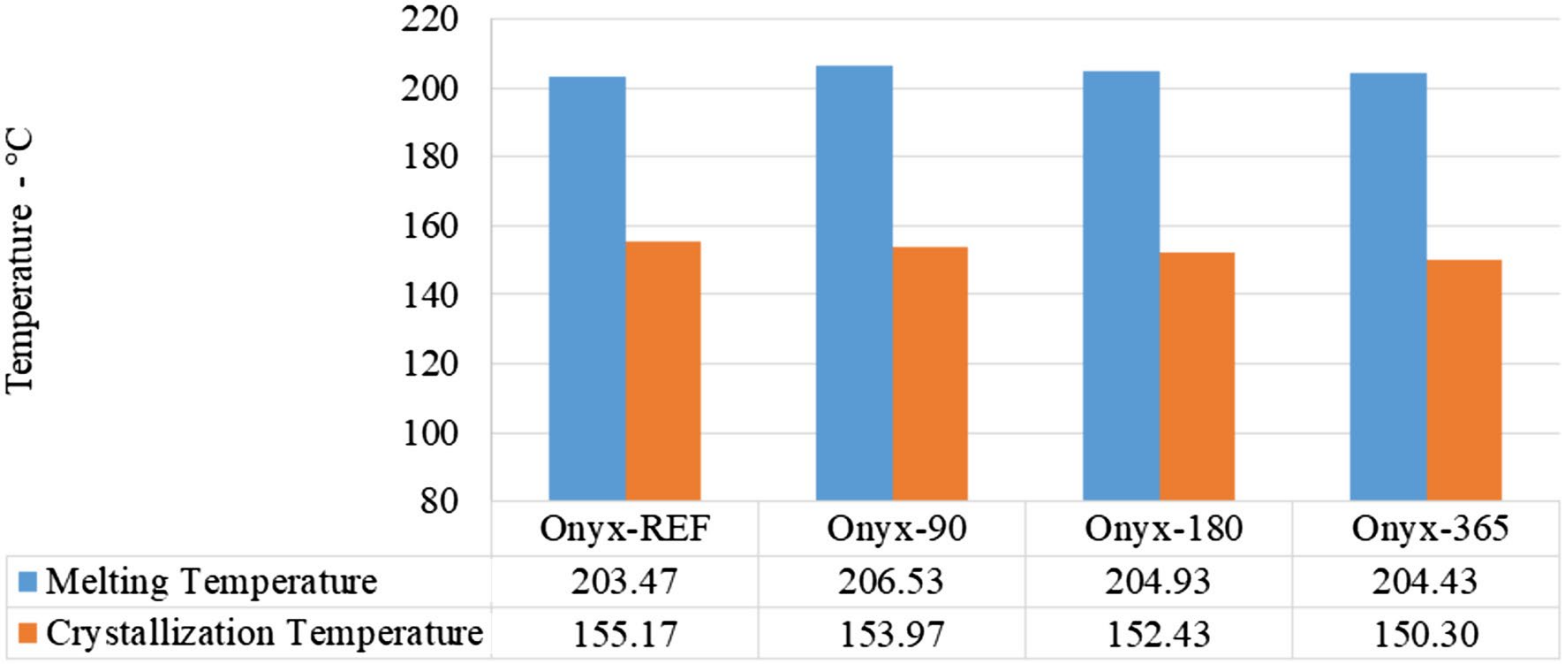
Change in melting point (MP) and crystallization temperature (CT) of aged specimens

The temperature scan comprised three stages: the first scan involved heating the samples from 40 to 250 °C, the second scan entailed cooling the samples from 250 back to 40 °C, and the final scan consisted of reheating the samples to 250 °C and subsequently cooling them to 40 °C. In each scan, the samples were held at 40 °C and 250 °C for a duration of 5 min. By analysing the experimental thermal results obtained from the scans, key thermal properties of the dry Onyx printed samples were determined. The melting point (MP) and crystallization temperature (CT) of the dry Onyx samples were calculated as 203 °C and 155 °C, respectively (Fig. 12).

Subsequently, the effect of aging in salt water on the thermal properties of Onyx was assessed. After 90 days of aging, the MP was measured as 206.5 °C, with a CT of 154 °C. Similarly, after 180 days of aging, the MP was found to be 205 °C, accompanied by a CT of 152.5 °C. The samples aged for 365 days exhibited an MP of 204.5 °C and a CT of 150 °C. Interestingly, these results indicate that the aging process had no significant impact on the thermal properties of the Onyx material, as the MP and CT values remained relatively stable throughout the aging period. These findings suggest that the structural integrity and thermal stability of the Onyx samples were maintained even after prolonged exposure to salt water. The lack of significant changes in the MP and CT values indicates that the material’s crystalline structure and thermal behaviour were resilient to the aging process. Overall, the temperature scan performed using the DSC 214 Polyma system provided valuable insights into the thermal properties of Onyx. The analysis of the dry and aged samples revealed consistent MP and CT values, indicating the material’s robustness against the effects of aging in salt water. These results contribute to our understanding of the thermal performance and stability of Onyx for various applications.

## Conclusion

The main conclusions of this study are:

- The gradual increase in water content observed in all tested materials over time is attributed to their capacity for moisture absorption, a phenomenon known as hygroscopic behaviour, compounded by voids and unfilled areas present during printing. The Onyx-Kevlar and Onyx-Carbon samples took longer to reach their saturation points and had higher water content compared to Onyx and Onyx-Glass specimens.
- The relationship between the fibre volume fraction and the water content at saturation point is straightforward. The higher fibre volume fraction, the higher water content.
- The research findings demonstrate that the Onyx material experienced a gradual decline in tensile strength over time, indicating a sensitivity to prolonged exposure to saltwater, resulting primarily from mechanical degradation before reaching the saturation point. Despite a substantial initial loss of strength (30%) within 90 days, the material’s tensile strength remained relatively stable after a full year of saltwater exposure. conversely, Onyx composites reinforced with continuous fibres, such as Glass, Carbon, and Kevlar, exhibited considerable reductions in tensile strength, emphasizing their susceptibility to extended saline exposure, even after reaching saturation.
- Microscopic analysis through SEM images of fractured tensile samples revealed distinct failure modes within the materials. While the failure mode in the Onyx samples remained consistent at the overlap of printing beads throughout the aging process, composites reinforced with continuous fibres exhibited primary failure modes involving fibre breakage and debonding between fibres and the thermoplastic. Fibre breakage emerged as the primary failure mode in aged samples, indicating the impact of extended exposure to moisture on structural integrity.
- The investigation into the flexural strength of additively manufactured composites exposed to saltwater aging highlighted significant reductions in strength over time. These findings underscore the need to consider long-term performance and durability limitations of these materials in real-world marine applications. Moisture absorption initiated complex processes, such as plasticization, swelling, matrix hydrolysis, and interfacial debonding, ultimately contributing to a gradual decline in mechanical properties.
- The mechanical strength degradation during the aging process was more pronounced in pure Onyx samples compared to Onyx reinforced with continuous fibres. Pure Onyx showed significant reductions in both tensile (33.54%) and flexural (63.47%) strengths after a year, while continuous fibre-reinforced Onyx exhibited lower strength reductions. The Onyx + Glass samples had the smallest reduction in tensile strength, while Onyx + Carbon showed the least reduction in flexural strength. Conversely, Onyx + Kevlar and Onyx Carbon demonstrated the highest reduction in tensile strength (approximately 28.5%).
- The indentation testing provided valuable insights into the microscale properties of Onyx. The modulus of elasticity of dry Onyx measured at the microscale was slightly higher than the value obtained from standard tensile testing.
- The indentation technique revealed higher modulus of elasticity values at the microscale compared to macro-level measurements, emphasizing its sensitivity to micro variations in material properties. Additionally, the micro-hardness of Onyx reduced over time during the aging process, signifying changes in the material’s hardness properties. It is worth noting that the micro-hardness of the aged specimens were higher than the un-aged Onyx sample indicating higher brittleness in the Onyx printed specimens after reaction with salt water.
- The thermal analysis results suggest Onyx’s resilience to prolonged saltwater exposure, maintaining its crystalline structure and thermal stability.

## Data Availability

The data supporting this study’s findings are available on request from the corresponding author.
